# Maternal control of seed weight in rapeseed (*Brassica napus* L.): the causal link between the size of pod (mother, source) and seed (offspring, sink)

**DOI:** 10.1111/pbi.13011

**Published:** 2018-11-28

**Authors:** Na Li, Dongji Song, Wei Peng, Jiepeng Zhan, Jiaqin Shi, Xinfa Wang, Guihua Liu, Hanzhong Wang

**Affiliations:** ^1^ Oil Crops Research Institute of the Chinese Academy of Agricultural Sciences Key Laboratory of Biology and Genetic Improvement of Oil Crops Ministry of Agriculture Wuhan Hubei Province China; ^2^ Zhengzhou Fruit Research Institute of the Chinese Academy of Agricultural Sciences The Laboratory of Melon Crops Zhengzhou Henan Province China

**Keywords:** *Brassica napus*, *CYP78A9*, maternal effect, photosynthesis, pod length, quantitative trait locus, RNA‐seq, seed weight, sink, source

## Abstract

Seed size/weight is one of the key traits related to plant domestication and crop improvement. In rapeseed (*Brassica napus* L.) germplasm, seed weight shows extensive variation, but its regulatory mechanism is poorly understood. To identify the key mechanism of seed weight regulation, a systematic comparative study was performed. Genetic, morphological and cytological evidence showed that seed weight was controlled by maternal genotype, through the regulation of seed size mainly via cell number. The physiological evidence indicated that differences in the pod length might result in differences in pod wall photosynthetic area, carbohydrates and the final seed weight. We also identified two pleiotropic major quantitative trait loci that acted indirectly on seed weight via their effects on pod length. RNA‐seq results showed that genes related to pod development and hormones were significantly differentially expressed in the pod wall; genes related to development, cell division, nutrient reservoir and ribosomal proteins were all up‐regulated in the seeds of the large‐seed pool. Finally, we proposed a potential seed weight regulatory mechanism that is specific to rapeseed and novel in plants. The results demonstrate a causal link between the size of the pod (mother, source) and the seed (offspring, sink) in rapeseed, which provides novel insight into the maternal control of seed weight and will open a new research field in plants.

## Introduction

Seed size/weight is one of the key traits related to plant fitness and crop yield (Gnan *et al*., 2014). Seed size/weight shows extensive natural variation even within the different accessions of a given species, which is of great value for evolution/domestication research and crop improvement. However, the regulatory mechanism of this kind of natural variation is poorly understood.

From a genetic and developmental perspective, seed is the heterotrophic organ resulting from double fertilization. It comprises three parts with different genetic compositions: diploid embryo (zygotic tissue), triploid endosperm (zygotic tissue) and diploid seed coat (maternal tissue). In addition, seed is formed in the mother plant inside the fruit, and thus seed development is controlled by the interaction of three seed compositions and maternal plant tissues (Alonso‐Blanco *et al*., 1999). Therefore, seed traits (including seed size and quality, etc.) are theoretically determined by both maternal (nuclear and cytoplasmic) and zygotic (embryo and endosperm) effects. The genetic analyses of reciprocal crosses in several species, including *Arabidopsis* (Alonso‐Blanco *et al*., 1999), maize (Seka and Cross, 1995; Zhang *et al*., 2016), wheat (Minhas *et al*., 2014), cotton (Pahlavani and Abolhasani, 2006), peanut (Hariprasanna *et al*., 2008), sorghum (Mohammed *et al*., 2015), pea (Lemontey *et al*., 2000) and bean (Duc *et al*., 2001; Singh *et al*., 2017), have shown that both maternal and xenia (via zygote) effects have significant roles on seed size/weight. However, the relative importance of maternal and zygotic effects has not been analyzed and thus is unclear in rapeseed, in spite of its fundamental interest and significance in genetic study and breeding.

Physiologically, seeds act as sinks for assimilates from maternal source (Alonso‐Blanco *et al*., 1999). Seed weight, especially seed filling, ultimately depends on the availability of carbohydrates produced in the photosynthetic organ (source) and subsequent transport (translocation) to developing seeds (Zuo and Li, 2014). Recent studies have identified tens of genes and several pathways/mechanisms that underlie seed weight control, especially in model plants *Arabidopsis* and rice (Kesavan *et al*., 2013; Li and Li, 2015; Sundaresan, 2005; Zuo and Li, 2014). However, almost all of this progress has been restricted to the development of embryo, endosperm and seed coat, that is, the seed itself (sink), and/or the transport/partition of assimilates (translocation). In addition, most of these seed weight genes were identified using reverse genetics (mainly through mutant analysis), which cannot explain natural variation. Therefore, the regulation and relative contribution of (maternal) sources in determining the natural variation of seed weight remains poorly understood. Although green leaves and stems have been generally considered the major source of photosynthate, reproductive organs also have photosynthetic activity. In *Brassica*, seeds are contained in the pod/fruit wall, which also serves as a source of nutrients. After flowering, leaf senescence starts, the functional leaf area decreases rapidly (Allen *et al*., 1971), and the pod wall becomes an important site of CO_2_ fixation to support fruit growth (Lewis and Thurling, 1994; Mogensen *et al*., 1997). However, the relative contribution of pod wall photosynthesis to seed filling and the final seed weight remains unclear.

In the current study, we aimed to identify the key mechanism underlying the natural variation of seed weight in rapeseed (*Brassica napus* L.). We chose one pair of large‐ or small‐seed recombinant inbred lines (RILs) to perform a comparative study. We investigated the genetic, physiological, cytological and molecular causes of seed weight variation and found a causal link between the size/weight of seed (sink) and the length/area of the pod wall (source). Based on this experimental evidence, we proposed a potential seed weight regulatory mechanism that is specific to rapeseed and novel in plants. The results provide further insight into the maternal control of seed weight in rapeseed and thus offer potential solutions for its improvement.

## Results

### Seed weight and other agronomical and quality traits for large‐ or small‐seed RILs

The large F_2_ population of 1150 individuals showed a broad variation with thousand‐seed weight ranging from 2.28 to 6.93 g. From this F_2_ generation, according to successive directional selection (only via seed weight), the seed weight of screened RILs gradually stabilized. The thousand‐seed weight for the ten large‐ or small‐seed RILs at the F_8_ to F_9_ generation varied from 5.39 to 7.02 g and 2.60 to 3.76 g, with an average of 6.16 and 3.12 g respectively (Table S1).

Interestingly, pod length showed a similar trend to thousand‐seed weight, and it was all significant larger (*P* = 1.22E‐12) in large‐seed RILs than small‐seed ones (38.2%). In view of the trade‐off between seed weight and seed number (Zhang *et al*., 2007), we also investigated seed number per pod and seed yield per pod. Although seed number per pod for large‐seed RILs was usually smaller than that of small‐seed RILs (24.6%), seed yields per pod for large‐seed RILs were usually larger than that for small‐seed RILs (39.1%). Moreover, the protein content of large‐seed RILs was usually larger than that for small‐seed RILs (13.4%) (Table S1).

To further investigate the probable cause of seed weight difference between these RILs, one pair of the large‐ and small‐seed RILs (L_RIL_0974_ and S_RIL_1148_) that showed similar flowering times were selected for subsequent research.

### Maternal genotype controls seed weight through the regulation of its size mainly via cell number

**Figure 1:**
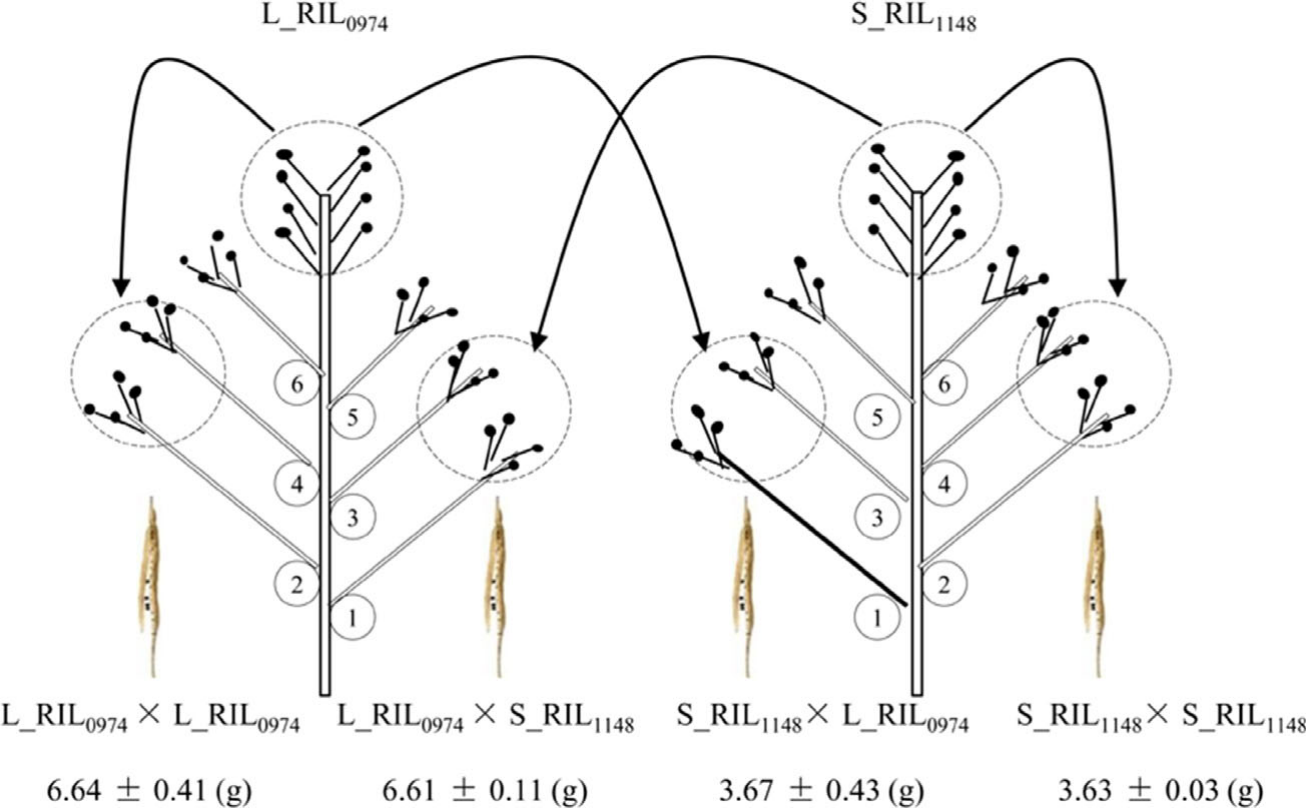
Maternal genotype controls the seed weight. Thousand‐seed weight was determined in self‐fertilized and cross‐fertilized seeds from the same plants. Data represent means of at least four independent plants.

First, the genetic basis of the seed weight difference between L_RIL_0974_ and S_RIL_1148_ was investigated by a self‐ or cross‐pollination experiment (Figure 1). The F_1_ inbred and hybrid seeds developed on L_RIL_0974_ (6.64 and 6.61 g) and S_RIL_1148_ (3.63 and 3.67 g) mother plant showed no significant difference in thousand‐seed weight (Table 1), whereas the former was significantly heavier than the latter. The calculated maternal effects on seed weight were 0.97 and 0.88, respectively, for L_RIL_0974_ and S_RIL_1148_, which were similar to the values estimated using other rapeseed lines (Li *et al*., 2015), indicating a dominant role of maternal parent in determining seed weight. In the F_2_ generation, seeds from the F_1_ hybrid plant of two reciprocal (L_RIL_0974_ × S_RIL_1148_ and S_RIL_1148_ × L_RIL_0974_) crosses also showed no significant difference in thousand‐seed weight (3.49 and 3.94 g, *P* = 0.69) and were similar to the results obtained from other rapeseed lines (Li *et al*., 2015), indicating an absent or small cytoplasmic effect on seed weight. Taken together, our results strongly suggest the natural variation of seed weight is mainly controlled by maternal genotype.

**Table 1:** Morphological and cytological traits in L_RIL_0974_, S_RIL_1148_ and their reciprocal crosses

Material	Thousand‐ seed weight (g)	Seed diameter (mm)	Seed surface area (mm^2^)	Seed volume (mm^3^)	Bulk density (mg/mm^3^)	Seed coat cell size (μm^2^)	Seed coat cell number	Cotyledon cell size (μm^2^)	Cotyledon cell number
L_RIL_0974_ × L RIL_0974_	6.64 ± 0.41a	2.19 ± 0.05a	15.08 ± 0.66a	5.51 ± 0.36a	1.21 ± 0.12a	329.57 ± 46.11ab	46 681 ± 7804a	656.52 ± 86.59a	23 359 ± 3482a
L_RIL_0974_ × S_RIL_1148_	6.61 ± 0.11a	2.19 ± 0.03a	15.08 ± 0.42a	5.51 ± 0.23a	1.20 ± 0.05ab	334.98 ± 26.90a	45 279 ± 3730a	646.83 ± 50.00ab	23 429 ± 1711a
S_RIL_1148_ × S_RIL_1148_	3.63 ± 0.03b	1.81 ± 0.01c	10.24 ± 0.06c	3.08 ± 0.03b	1.18 ± 0.05ab	300.55 ± 23.07b	34 249 ± 2761b	587.05 ± 65.99b	17 662 ± 2219b
S_RIL_1148_ × L_RIL_0974_	3.67 ± 0.43b	1.84 ± 0.03b	10.60 ± 0.35b	3.25 ± 0.16b	1.13 ± 0.10b	321.44 ± 36.56ab	33 374 ± 3923b	638.06 ± 75.97ab	16 830 ± 2033b

Columns followed by the same letter are not significantly different at *P* < 0.05 (Duncan's test).

Second, the morphological basis of seed weight difference was investigated (Table 1). The bulk density showed no significant difference between L_RIL_0974_ and S_RIL_1148_. However, the F_1_ seed of L_RIL_0974_ × L_RIL_0974_ was significantly larger than that of S_RIL_1148_ × S_RIL_1148_ in diameter, surface area and volume, with the proportions of 21.0%, 47.3% and 78.9% respectively. The calculated maternal effect values of seed diameter/surface area/volume were 1.00/0.99/0.99 and 0.92/0.93/0.93, respectively, for L_RIL_0974_ and S_RIL_1148._

Third, the cytological basis of seed size difference was investigated (Table 1). The F_1_ seed of L_RIL_0974_ × L_RIL_0974_ and S_RIL_1148_ × S_RIL_1148_ differed significantly in cell number of seed coat and cell number and size of cotyledon, but not in cell size of seed coat. Nevertheless, cell number should be the major factor contributing to the seed size difference between them, because the former had 36.3% and 32.3% more cells than the latter in seed coat and cotyledon, respectively, whereas these cells were only 9.7% and 11.8% larger. Similarly, the F_1_ seed of L_RIL_0974_ × S_RIL_1148_ and S_RIL_1148_ × L_RIL_0974_ differed significantly in cell number, but not in cell size; and the former had 35.7% and 39.2% more cells than the later in seed coat and cotyledon, respectively, whereas these cells were only 4.2% and 1.4% larger. The calculated maternal effect values of seed coat cell number/seed coat cell size/cotyledon cell number/cotyledon cell size were 0.78/0.61/0.82/0.49 and 0.99/0.36/1.00/0.78, respectively, for L_RIL_0974_ and S_RIL_1148._

### Photosynthate from pod wall is the major contributor to seed weight in rapeseed

**Figure 2:**
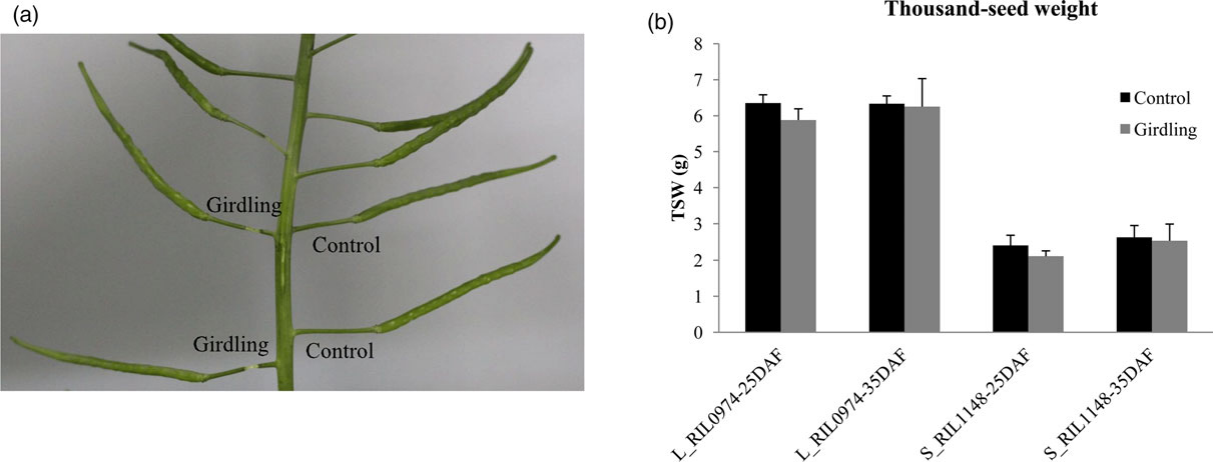
Assimilates from the pod wall contribute to seed weight. (a) A girdling experiment was performed. (b) Seed weight of girdling pod and controls at different stage. Values are means ± SE of three independent experiments.

The seed filling is dependent on the supply of photosynthate from maternal plant tissues, including leaves, green stems and pod/fruit wall in *Brassica* (Inanaga *et al*., 1979). During the reproductive phase, the rapid senescence of functional leaves and development of pods make its wall as the major source of photosynthate. To distinguish the roles played by the pod wall or other source organs for seed filling, we performed girdling experiments at 25 and 35 days after flowering (DAF) by removal of the pedicel phloem (Figure 2a). The results showed that the seed weight of both L_RIL_0974_ and S_RIL_1148_ was reduced after girdling treatments, while the proportion decreased from 8.0% to 14.2% (25‐DAF) to 1.3% and 3.2% (35‐DAF) respectively. However, the seed weight difference between girdling treatment and control was not significant for either L_RIL_0974_ (*P* = 0.06 and 0.80) or S_RIL_1148_ (*P* = 0.13 and 0.17) at the two stages (Figure 2b). These results suggest that the pod wall might be the main source of photosynthate for seed filling, that is, pod photosynthate is the major contributor to final seed weight in rapeseed.

### Pod wall photosynthetic area is highly associated with seed weight

**Figure 3:**
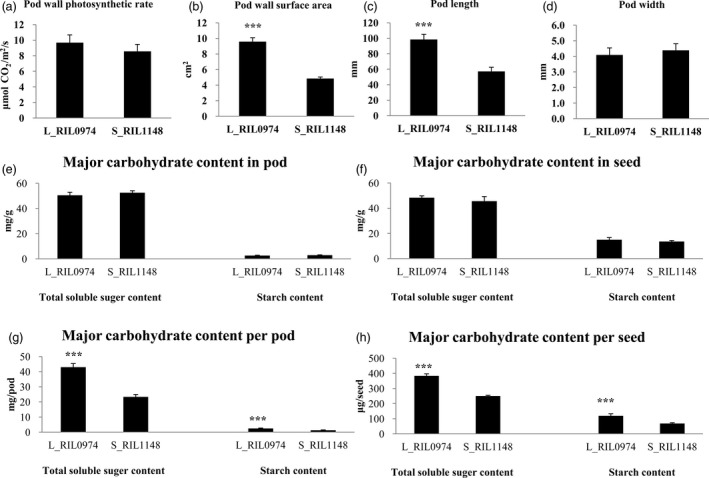
Pod wall photosynthesis associated traits (a, b, c and d) and major carbohydrate contents, including total soluble sugar and starch, in the pod wall and developing seeds (e, f, g and h). (a) pod wall photosynthetic rate; (b) pod wall surface area; (c) pod length; and (d) pod width; (e) major carbohydrate content in pod; (f) major carbohydrate content in seed; (g) major carbohydrate content per pod; and H: major carbohydrate content per seed. “***” represents extremely significant at *P* < 0.001. Values are means ± SE of three independent experiments.

To assess the role of pod wall photosynthesis in the formation of the seed weight difference, we measured the photosynthetic rate and area of the pod wall at 25‐DAF, the time at which surface area and apparent photosynthesis of the pod wall both reached the maximum value (Inanaga *et al*., 1979). The results showed that the pod wall photosynthetic rates of the L_RIL_0974_ and S_RIL_1148_ were similar, whereas the pod wall area of the former was significantly larger than that of the latter, with a proportion of 97.7% (Figure 3a,b). These results implied a link between pod wall area (source) and seed size (sink). We further compared the pod length and width and found the pod widths of the two RILs were similar, whereas the pod length of the former was significantly longer than that of the latter, with a proportion of 72.0% (Figure 3c,d).

To investigate whether the difference in pod wall area had an effect on the photosynthetic product synthesis in the pod wall and its storage in seed, major carbohydrate contents and gross, including total soluble sugar and starch, in the pod wall and developing seeds were determined. The results showed that total soluble sugar and starch contents in the pod wall and developing seed of L_RIL_0974_ and S_RIL_1148_ showed no significant difference (Figure 3e,f). However, the total soluble sugar and starch gross in both pod wall and developing seeds of L_RIL_0974_ were higher than those of S_RIL_1148_ (Figure 3g,h) because of their higher fresh weight.

These results suggest that the pod length difference between L_RIL_0974_ and S_RIL_1148_ resulted in the difference in pod wall photosynthetic area and the following photosynthetic product synthesis in the pod wall and its storage in seed, which led to the difference in the final seed weight.

### Pod length could contribute to seed weight via a maternal effect: evidence from two major pleiotropic QTLs

To find molecular evidence for the causal link between pod length and seed weight, we performed QTL mapping and comparison using the BnaZNRIL population (Shi *et al*., 2015). Both seed weight and pod length of the population showed normal or near‐normal distributions at two locations over two successive years (Figure S1), suggesting a quantitative inheritance pattern. The broad‐sense heritability of seed weight and pod length was 0.85 and 0.93, respectively, which were high and consistent with previous studies (Zhang and Zhou, 2006). In addition, a significant positive correlation was found between these two traits in all of the investigated year‐location combinations (mean *r* ^2^ = 0.45; *P* < 0.001).

**Figure 4:**
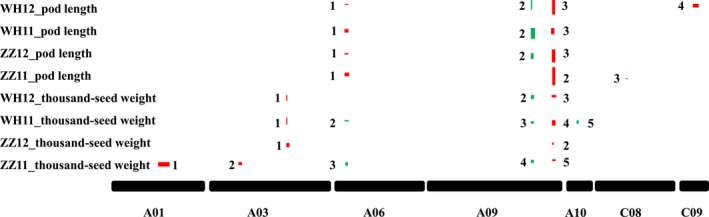
Localization of QTLs for seed weight and pod length. Box widths indicate LOD 2.0 confidence intervals for the QTLs. The box height represents the percentage variance explained. Red indicates the positive effect of Zhongshuang11 allele, green indicates that of No. 73290. ZZ and WH denote Zhengzhou and Wuhan respectively.

A skeleton linkage map of 19 linkage groups and 2174 markers (mainly SNPs (98.7%), the rest were SSRs and InDels) was constructed (Table S2), which covered a total length of 2155 cm, with an average distance of approximately 1 cm between adjacent markers. We scanned genome‐wide QTLs for thousand‐seed weight and pod length respectively (Table S3). A total of 15 QTLs for thousand‐seed weight were mapped; seven in Zhengzhou (five in 2011 and two in 2012) and eight in Wuhan (five in 2011 and three in 2012). For pod length, six QTLs were identified in Zhengzhou (three in 2011 and three in 2012) and seven in Wuhan (three in 2011 and four in 2012). Three colocalized QTL pairs (one on A06 and two on A09) were identified for both traits (Figure 4) and explained a large proportion of the variation (total *R* ^2^ = 19.0% and 53.9% for thousand‐seed weight and pod length respectively), especially for pod length, in accordance with the strong genetic correlation observed between these two traits.

**Figure 5:**
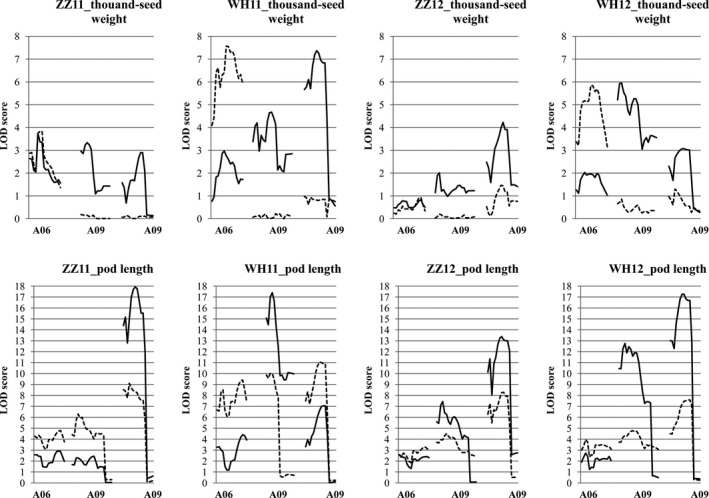
LOD profiles comparing conditional (dotted line) and unconditional (solid line) QTL scans for seed weight and pod length. ZZ and WH denote Zhengzhou and Wuhan, respectively.

Genetically, QTL colocalization is considered to be caused by either tight linkage or pleiotropy (Wagner and Zhang, 2011). To determine the genetic causations for the three colocalized QTL pairs for both traits (Figure S2 **)**, conditional QTL analysis was performed (Figure 5 and Table S3). For the colocalized QTLs on A06, regardless of whether thousand‐seed weight was conditioned by pod length or pod length was conditioned by thousand‐seed weight, both showed similar effects in the unconditional analysis, indicating a genetic cause of tight linkage rather than pleiotropy. Both the thousand‐seed weight QTLs on A09 disappeared when thousand‐seed weight was conditioned by pod length. However, when pod length was conditioned by thousand‐seed weight, both pod length QTLs on A09 were still significant. These results suggest that pleiotropy was the genetic basis for the two genomic regions for both traits, that colocalized QTLs act indirectly on thousand‐seed weight via their effects on pod length. In fact, one of the candidate genes for the two pleiotropic major QTLs has been identified in our lab (Liu *et al*., 2015), which also affects both seed weight and pod length. Furthermore, genome‐wide conditional analysis identified six new significant QTLs (two for thousand‐seed weight and four for pod length) on A03, A09, C01, C05, C06 and C09 respectively. These results suggest a genetic basis to thousand‐seed weight that does not entirely overlap with that of pod length.

### Transcriptome of the pod wall and seed

To obtain more information about how the maternal organ pod wall regulates final seed weight, two contrasting pools compared (large‐ vs. small‐seed RILs) at the transcriptome level. RNA‐seq generated 63 403 490 (pod wall from large‐seed pool), 62 866 348 (pod wall from small‐seed pool), 67 329 370 (seed from large‐seed pool) and 67 751 350 (seed from small‐seed pool) clean reads after filtering. The Phred quality score Q30 was >94% and the guanine‐cytosine (GC) content was basically 46% for each sample, suggesting high‐quality sequencing. Approximately 74.41%–77.14% of the clean reads were successfully mapped to the reference genome, and 57.95%–59.64% of them matched to unique genomic locations (Table S4).

A total of 631 (131 up‐ and 500 down‐regulated) and 486 (256 up‐ and 230 down‐regulated) differentially expressed genes (DEGs) were found for pod wall and seed respectively. To validate the results of gene expression profiles, we randomly selected 11 genes (schemed as 14 combinations) for quantitative real‐time PCR (qRT‐PCR, Table S5). Although the differences in gene expression did not entirely match the magnitude of these detected by Solexa‐based sequencing, the trend of up‐ and down‐regulation was similar (Figure S3). Solexa sequencing is more sensitive for estimation of gene expression (Wu *et al*., 2010). Given the same variation in expression profiles were observed with both methods, the transcriptome results were deemed to be reliable.

### DEGs in pod wall are highly associated with pod development

To provide a functional overview of the 631 DEGs in the pod wall (Table S6a), they were classified using the web‐tool SuperViewer (http://bar.utoronto.ca/ntools/cgi-bin/ntools_classification_superviewer.cgi), which showed the absolute number and relative frequency as well as the significance of *P*‐value for each class. Of the 16 significantly enriched classes (*P* < 0.05), nine extremely significantly enriched classes (*P* < 0.001), including misc, protein, cell wall, secondary metabolism, hormone metabolism, development, lipid metabolism, amino acid metabolism and DNA, reflected the differential developmental and metabolic status of the pod wall for two contrasting pools (Figure S4; Table S6b). Of the 77 DEGs grouped into the largest class of misc genes, 18 belonged to the cytochrome P450 family (Table S6a), which shared the highest proportion, suggesting the importance of cytochrome P450 in pod development. Most of the *Arabidopsis* orthologs of the 18 DEGs are involved in hormone metabolism, followed by secondary metabolism and lipid metabolism. In fact, the three metabolism groups were also present among the extremely significantly enriched groups, which suggested that they might be the downstream of cytochrome P450 genes. The 39 DEGs within the hormone metabolism class belonged to seven subclasses: JA synthesis/degradation (4), signal transduction (5) and response (1); AUX synthesis/degradation (3), response (3) and signal transduction (1); GA synthesis/degradation (3) and response (3); ABA synthesis/degradation (5) and response (1); ETH synthesis/degradation (3) and signal transduction (2); BR synthesis/degradation (2) and signal transduction (1); and CTK synthesis/degradation (3). These regults suggest the complex role of phytohormones in pod development. Of the 41 secondary‐metabolism DEGs, nearly half (18) were involved in metabolic processes of flavonoids, followed by glucosinolates, isoprenoids, simple phenols, phenylpropanoids and alkaloid, which suggested the importance of flavonoid metabolism in pod development. The other significantly enriched classes, such as lipid metabolism, protein, cell wall, amino acid metabolism and DNA, provide a material and structural basis for cell expansion and proliferation.

To establish the link between these DEGs and pod development, the fruit size genes (mostly from *Arabidopsis*) were collected and functionally classified (Table S6c). Two DEGs, *BnaA09g55530D* and *BnaA08g23390D*, are the orthologs of *Arabidopsis CYP78A9* and *CYP72C1*, respectively (Table S6a), which are positive and negative regulators of pod length (Sotelo‐Silveira *et al*., 2013; Takahashi *et al*., 2005). Interestingly, *BnaA09g55530D* and *BnaA08g23390D* were, respectively, up‐ and down‐regulated in the pool of large‐seed RILs compared with that of the small‐seed RILs, which was highly concordant with the longer pod of large‐seed RILs than small‐seed ones. In addition, more than half of the DEGs (62.0%) were involved in the functional categories/pathways of the collected fruit size genes, including phytohormone, transcription factor, protein synthesis, post‐translational modification and degradation (mostly by ubiquitin‐proteasome system), receptor kinase and G‐protein signalling, cell organization and cell wall proteins (Table S6a,c).

### DEGs in seed are associated with seed metabolism and development

The functional overview of the 486 DEGs in seed (Table S7a) was also analysed using the web‐tool SuperViewer. Of the 14 significantly (*P* < 0.05) enriched functional classes, eight classes (including misc, development, cell wall, transport, lipid metabolism, secondary mechanism, photosynthesis and DNA) were highly significantly (*P* < 0.001) enriched (Figure S5; Table S7b). Of the 42 DEGs grouped into the largest class of misc, myrosinases were most numerous (10), followed by cytochrome P450 (7), plastocyanin (5), invertase (3) and oxidases (3). Of the 21 secondary‐metabolism DEGs, more than half (10) were involved in glucosinolate degradation (most as myrosinases), followed by that of phenylpropanoids (3), alkaloid (3), flavonoids (3) and isoprenoids (2). These results suggest the importance of myrosinases in seed metabolism. Two functional classes, cell wall and DNA synthesis, provided the structural and material basis for cell proliferation and expansion. Interestingly, 11 DEGs were grouped into the light reaction of photosynthesis class, which suggested differences in photosynthesis in the seed coat between the two contrasting pools. Other predominant functional classes, including lipid metabolism, transport and development, reflected the differential ability of synthesis, transport and storage in the seeds of two contrasting pools.

Moreover, DEGs involved in development, cell division and nutrient reservoir activity were all up‐regulated in the large‐seed pool (Table S8). Of the development‐associated genes, one DEG encoding the *FIE* protein, which is responsible for seed development (Luo *et al*., 2000), was up‐regulated in the large‐seed pool. DEGs encoding a cell cycle‐regulated microtubule‐associated protein, the cell division control protein *cdc2* (Hirayama *et al*., 1991), *AtGRF5* (growth‐regulating factor 5) (Horiguchi *et al*., 2005) and a kinetochore protein were identified in the large‐seed pool.

To establish the link between these DEGs and seed development/size, the seed weight genes (mostly from *Arabidopsis*) were also collected and functionally classified (Table S7c). Notably, 55.3% of the DEGs were involved in the known pathways of seed size regulation, such as phytohormone, transcription factor, G‐protein and receptor kinase signalling, lipid metabolism, ubiquitin‐proteasome system, cytochrome P450 and cell cycle and division (Table S6a,c). More importantly, a total of 11 DEGs were homologous to known seed weight genes in *Arabidopsis*, including *AEP4*,* AGL62*,* CHS*,* CRA1*,* CRB*,* EMS1* and *ETO1*.

### *CYP78A9* participates in the interaction between pod wall and seed

The *CYP78A* subfamily is a plant‐specific cytochrome P450 family, some members of which regulate organ and plant size through cell proliferation and/or expansion in a non‐cell‐autonomous manner, e.g. *CYP78A5* in *Arabidopsis* (Adamski *et al*., 2009) and tomato (Chakrabarti *et al*., 2013), *CYP78A6* (Fang *et al*., 2012) and *CYP78A9* in *Arabidopsis* (Ito and Meyerowitz, 2000; Sotelo‐Silveira *et al*., 2013), *CYP78A11* in rice (Miyoshi *et al*., 2004), *CYP78A27* and *CYP78A28* in moss (Katsumata *et al*., 2011), *CYP78A10* and *CYP78A72* in soybean (Wang *et al*., 2015; Zhao *et al*., 2015) and *CYP78A3* in wheat (Ma *et al*., 2015). Because *CYP78A* expression is always inconsistent with proliferating and/or expanding regions, where mutant phenotypes are observed in *Arabidopsis* and rice, *CYP78A* most likely acts in generating a novel mobile signal controlling cell proliferation, which implies potential links between different organs, such as pod wall and seed. Several DEGs homologs to *Arabidopsis* cytochrome P450 gene were observed in our transcriptome analysis of the pod wall and seed (Table 2). *BnaA09g55530D* (homologous to *Arabidopsis CYP78A9*) was the only DEG in both pod wall and seed. However, the expression level of *CYP78A9* in the pod wall was significantly higher than that in seed. Much research has been performed on *CYP78A9* in *Arabidopsis* (Ito and Meyerowitz, 2000; Sotelo‐Silveira *et al*., 2013). The results show that mutants of *CYP78A9* have short pods and small seeds, while overexpression of *CYP78A9* induces large and seedless pods (the minority produce larger seeds in comparison with the wild‐type). In addition, there may exist a novel mobile signal that is independent of known phytohormones coordinating growth between sporophytic and gametophytic tissue and between the structures that protect the ovules and the seed. Both the observed phenotypes of *CYP78A9* overexpression and knockout and the *CYP78A9*‐specific expression pattern suggest a link (*CYP78A9*‐produced mobile signal) between pod (source) and seed (sink) size. Furthermore, a comparison was made between previous microarray analysis of transcriptional responses to *CYP78A9* (Sotelo‐Silveira *et al*., 2013) and the current RNA‐seq analysis in the pod wall. Interestingly, 23 down‐regulated and two up‐regulated genes were identified in both experiments (Table 3). In addition, the expression levels of six genes were inconsistent with each other between the microarray analysis of transcriptional responses to *CYP78A9* and our analysis. These results suggest that a *CYP78A9*‐produced mobile signal pathway might exist.

**Table 2:** List of DEGs identified in the pod wall of two contrasting pools that are homologous to CYP 450 family genes

*B. napus* gene ID	Small seed pool‐RPKM	Large seed pool‐RPKM	log_2_ Ratio(large seed pool/small seed pool)	Up‐down‐regulation (large seed pool/small seed pool)	Annotation
*BnaA03g25800D*	2.83	0	−Inf	Down	*CYP702A1*
*BnaA03g33480D*	2.04	0	−Inf	Down	*CYP72A9*
*BnaA03g35180D*	7.3	18.46	1.34	Up	*CYP707A4*
*BnaA03g57690D*	8.25	26.55	1.69	Up	*CYP82G1*
*BnaA04g24160D*	9.18	18.88	1.04	Up	*CYP83A1*
*BnaA05g12840D*	20.72	9.52	−1.12	Down	*CYP81D1*
*BnaA06g16470D*	16.8	6.33	−1.41	Down	*CYP94B3*
*BnaA06g31960D*	3.28	0	−Inf	Down	*CYP96A12*
*BnaA06g32050D*	5.73	0	−Inf	Down	*CYP709B3*
*BnaA07g13320D*	14.62	6.31	−1.21	Down	*CYP94C2*
*BnaA08g23390D*	14.44	0	−Inf	Down	*CYP72C1*
*BnaA09g15160D*	26.11	9.34	−1.48	Down	*CYP96A15*
*BnaA09g21050D*	18.02	43.41	1.27	Up	*CYP706A4*
*BnaA09g28210D*	2.49	0	−Inf	Down	*CYP86C1*
*BnaA09g55530D*	32.33	237.76	2.88	Up	*CYP78A9*
*BnaC03g56910D*	4.67	0	−Inf	Down	*CYP71B23*
*BnaC04g16670D*	6.98	2.25	−1.63	Down	*CYP94C1*
*BnaC05g20990D*	2	0	−Inf	Down	*CYP86C2*
*BnaC07g16020D*	13.13	6.29	−1.06	Down	*CYP81G1*
*BnaC07g35800D*	16.81	6.02	−1.48	Down	*CYP707A1*
*BnaC09g23280D*	8.8	22.46	1.35	Up	*CYP706A4*
*BnaCnng02380D*	11.96	5.38	−1.15	Down	*CYP86A2*

**Table 3:** List of common DEGs between the previous microarray analysis of transcriptional responses to *CYP78A9* and our RNA‐seq analysis in the pod wall

*B. napus* gene ID	*A. thaliana* ortholog	Current Transcriptome analysis in pod wall^a^	Transcriptional responses to *CYP78A9* b	Annotation
*BnaA01g16400D*	*AT4G27410*	Down (−1.89)	Down (−2.98)	NAC‐domain protein 485
*BnaA01g19500D*	*AT3G51370*	Down (−2.85)	Down (−2.06)	Protein phosphatase 2c‐like protein
*BnaA04g00590D*	*AT2G46400*	Up (1.18)	Down (−2.26)	*WRKY46*
*BnaA04g24790D*	*AT2G43050*	Down (−4.97)	Down (−2.17)	Pectinesterase family protein
*BnaA05g12840D*	*AT5G36220*	Down (−1.12)	Down (−2.26)	*CYP81D1*
*BnaA06g14440D*	*AT1G20500*	Down (−4.16)	Down (−3.28)	4‐coumarate‐CoA ligase
*BnaA06g34530D*	*AT2G01170*	Down (−1.45)	Down (−2.10)	Amino acid or GABA permease
*BnaA06g36830D*	*AT1G27730*	Down (−1.48)	Down (−2.16)	His2‐type zinc finger protein 3
*BnaA07g17630D*	*AT2G46430*	Down (−Inf)	Down (−3.25)	Cyclic nucleotide gated ion channel 11
*BnaA07g22140D*	*AT1G74640*	Down (−1.21)	Down (−2.12)	Unknown protein
*BnaA07g28880D*	*AT1G70780*	Down (−1.05)	Down (−2.26)	Unnamed protein
*BnaA09g05560D*	*AT5G61000*	Up (1.49)	Up (2.19)	Replication protein A1
*BnaA09g55030D*	*AT3G54940*	Down (−4.60)	Down (−2.07)	Cysteine‐type peptidase
*BnaA10g20890D*	*AT5G14470*	Down (−4.45)	Down (−2.28)	GHMP kinase‐related
*BnaAnng05900D*	*AT5G64080*	Down (−1.69)	Down (−2.54)	Lipid transfer protein family protein
*BnaC02g00930D*	*AT5G64080*	Down (−2.53)	Down (−2.54)	Lipid transfer protein family protein
*BnaC02g04560D*	*AT5G13200*	Down (−1.08)	Down (−2.17)	ABA‐responsive protein‐related
*BnaC02g09510D*	*AT5G22430*	Down (−5.31)	Down (−3.26)	Unknown
*BnaC03g23150D*	*AT2G41140*	Up (1.06)	Down (−2.06)	*CRK1*
*BnaC04g21660D*	*AT2G46400*	Up (1.16)	Down (−2.26)	*WRKY46*
*BnaC05g01450D*	*AT1G02820*	Down (−1.47)	Down (−2.56)	Late embryogenesis abundant 3 family protein
*BnaC07g16020D*	*AT5G67310*	Down (−1.06)	Down (−2.84)	*CYP81G1*
*BnaC07g40860D*	*AT4G27410*	Down (−1.08)	Down (−2.98)	*RD26*
*BnaC08g26020D*	*AT3G55120*	Down (−3.45)	Down (−2.07)	Chalcone‐flavanone isomerase 1 protein
*BnaC09g05110D*	*AT5G61000*	Up (1.13)	Up (2.19)	Replication protein A1
*BnaC09g24180D*	*AT4G11210*	Down (−Inf)	Up (2.12)	Disease resistance‐responsive family protein
*BnaC09g51750D*	*AT1G58410*	Down (−1.56)	Up (2.30)	Disease resistance protein
*BnaCnng01610D*	*AT4G00780*	Down (−2.50)	Up (2.46)	MATH domain‐containing protein
*BnaCnng30140D*	*AT2G43050*	Down (−1.60)	Down (−2.17)	Pectinesterase family protein
*BnaCnng60000D*	*AT1G32350*	Down (−1.20)	Down(−2.24)	Alternative oxidase
*GSBRNA2T00046081001*	*AT5G62740*	Down (−Inf)	Down (−2.68)	Band 7 family protein

a The values in the brackets indicate the log_2_ Ratio(large seed pool/small seed pool;

b The values in the brackets indicate the Z‐score.

In our pathway enrichment analysis (Table S9a,b) in the pod wall, *BnaA09g55530D* participated in several metabolic pathways, including phenylpropanoids biosynthesis, flavonoid biosynthesis, limonene and pinene degradation, flavone and flavonol biosynthesis, stilbenoid, and diarylheptanoid and gingerol biosynthesis. The gene was up‐regulated in the transformations from dihydrokaempferol to dihydroquercetin, kaempferol to quercetin, apigenin to luteolin, naringenin to eriodictyol, liquiritigenin to butin, and garbanzol to dihydrofisetin (Figure S6). Other three DEGs homologous to the cytochrome P450 family (*CYP706A4* and *CYP82G1*) also participated in these pathways together with *CYP78A9*.

### Proposed regulation mechanism for the maternal control of seed weight in rapeseed

**Figure 6:**
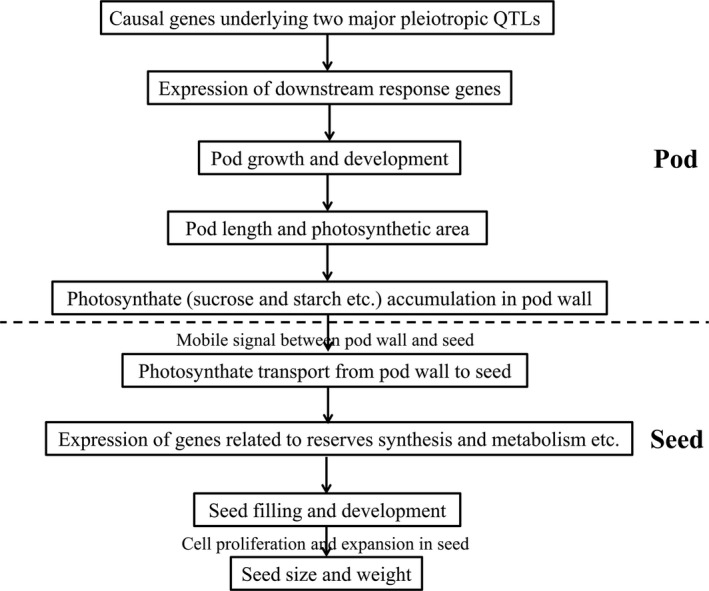
A proposed model for the maternal control of seed weight via pod.

According to the abovementioned experimental results, we proposed a potential regulation mechanism model for the maternal control of seed weight via the pod in rapeseed (Figure 6). For the two major pleiotropic QTLs, the variations of the underlying causal genes in protein function and/or expression level lead to changes in expression levels of the downstream response genes (such as *BnaA09g55530D*) related to pod growth and development, which result in changes in pod length, photosynthetic area and photosynthate gross in the pod wall. At the same time, the mobile signal between pod wall and seed also changes, affecting the transport of photosynthate from pod wall to seed and then influencing the expression of genes related to reserves synthesis and metabolism, which finally affect seed filling, size and weight.

## Discussion

Seed weight/size is a highly important trait contributing to plant fitness and crop yield, which has long been a research focus. Seed weight/size is also a very complex trait due to its heterotrophic genetic composition, which is controlled by both maternal and zygotic effects (Li *et al*., 2015). Although the relative contribution of maternal and zygotic effects on seed weight have fundamental importance in both theoretical and applied research (except for autogamous plants), it has not been studied and thus is unclear in rapeseed.

Consistent with our previous study using several representative rapeseed lines (Li *et al*., 2015), the seed weight difference between the two large‐ or small‐seed RILs was found to be controlled mainly by maternal genotype, which suggests it should to be a general rule. The further morphological and cytological analyses showed seed weight was determined by seed size rather than bulk density, and cell number was the major factor contributing to final seed weight, which was also in agreement with our earlier study (Li *et al*., 2015). Moreover, strong maternal effects have been observed for seed size and cell number in *Arabidopsis* (Alonso‐Blanco *et al*., 1999), providing more evidence that seed size and cell number are the main causes of the coordinate variation with seed weight. The influence of mother plant on sink is mainly expressed in the role of the source, that is, providing photoassimilate to developing seeds (Bravo *et al*., 1980). Meanwhile, the sink effect on source might, through feedback/feedforward regulation, control fertilization to initiate pod growth. In brief, source and sink are highly interdependent and are not truly a simple two‐component system (Egli and Bruening, 2001). The decline of functional leaf area and pod development results in a change of the main source (King *et al*., 1997). Our girdling experiment showed that pod wall photosynthate is the major contributor to final seed weight in rapeseed, which is consistent with a previous study (Mackay, 2004). The parallel timing of pod wall carbon loss and rapid seed growth has been observed (Norton and Harris, 1975). Therefore, in the grain filling stage, pod wall tissue could be the major supplier of carbon to developing seeds.

We found that differences in pod length altered the pod wall photosynthetic area, total carbohydrate gross of pod wall and seed and final seed weight. Pod length and width are significantly correlated with final seed size in soybean (Bravo *et al*., 1980). At the molecular level, two pleiotropic major QTLs for both seed weight and pod length have been identified in the current study and several other genetic populations (Fu *et al*., 2015; Li *et al*., 2014; Yang *et al*., 2012), which suggests that seed weight could have contributions from a mother resource(such as the pod). Comparative analyses suggested that 32 homologues of known fruit/seed size genes (Christians *et al*., 2009; Ehlers *et al*., 2016; Fang *et al*., 2011; Le Hir *et al*., 2015; Li *et al*., 2008, 2010; Liu and Makaroff, 2006; Liu *et al*., 2008; Mizoi *et al*., 2006; Ogawa‐Ohnishi and Matsubayashi, 2015; Ok *et al*., 2015; Riefler *et al*., 2006; Schruff *et al*., 2006; Steffen *et al*., 2008; Zuniga‐Sanchez *et al*., 2014) were located in the confidence intervals of QTLs for seed weight and pod length (Table S3). Of these, only *BnaA03g19500D and BnaA09g55580D* showed sequence variations between the two parents. Notably, *BnaA09g55580D* (*BnaA.ARF18*), which regulated both pod length and seed weight, has been validated and cloned in our lab (Liu *et al*., 2015). Moreover, several genes have been identified as affecting seed weight/size and the mother plants photosynthetic organs. *TGW6* has pleiotropic effects on grain weight and source organs (the accumulation of carbohydrates before heading) in rice (Ishimaru *et al*., 2013). Overexpression of rice *TIFY11b* enhances accumulation and translocation of carbohydrate in the culms and leaf sheaths, leading to increased grain size and weight (Hakata *et al*., 2012). Therefore, it seems possible that seed may sense the source availability, indicating a connection between the sizes of pod and seed, that is, the sizes of source and sink.

Of the total 1063 DEGs detected in pod wall and seed, only 54 DEGs overlapped between the data sets, which suggested different expression regulation modes between two tissues. Plant hormones (Walton *et al*., 2012) and several metabolite enzymes (Fang *et al*., 2012; Ito and Meyerowitz, 2000) play key roles in the fruit development. In the processes of signal transduction, signal molecules (including various hormones and specifically, enzymes) can produce mobile signals that play important roles in response to a broad spectrum of genes and complex biological pathways. In the present study, DEGs involved in signal transduction were overrepresented in the large‐seed pools of the pod wall. Of the up‐regulated genes, some hormone and secondary‐metabolite enzymes were identified. One such DEG, homologous to *CYP72C1*, controls BR homoeostasis by modulating BR concentration and is involved in BR degradation (Takahashi *et al*., 2005). Two DEGs homologous to *CYP707A* family genes encode abscisic acid 8′‐hydroxylase, involved in abscisic acid degradation (Okamoto *et al*., 2006, 2011). Four DEGs (all homologous to *Arabidopsis JAZ1*) encode jasmonate‐zim‐domain protein 1, involved in jasmonate signalling (Geerinck *et al*., 2010). One DEG homologous to *AtSOFL1* acts redundantly with *AtSOFL2* as a positive regulator of cytokinin levels and cytokinin‐mediated development (Zhang *et al*., 2009). Two DEGs, encoding cytokinin dehydrogenase, were down‐regulated in the small‐seed pools of the pod wall. We also found increased expression of *BnaA09g55530D* and its potential target genes in the large‐seed pools of the pod wall (Table 3). Interestingly, this DEG is located in the confidence interval of a pleiotropic major QTL for both pod length and seed weight, but it shows no sequence variation between the two parents. The abovementioned function and expression level analyses of *CYP78A9* in *Arabidopsis* (Ito and Meyerowitz, 2000; Sotelo‐Silveira *et al*., 2013) could well explain the expression of DEGs in the pod wall and seed, but the two major pleiotropic QTLs for pod length and seed weight have been fine‐mapped and/or cloned in our lab (Liu *et al*., 2015). These results suggest that *BnaA09g55530D* is likely to be an important downstream gene regulated by the major QTLs for both pod length and seed weight.

Our study provided a general genetic rule that the natural variation of seed weight is mainly controlled by maternal genotype in rapeseed. In this context, we conducted a systematic research (encompassing morphological and cytological analysis, physiological and biochemical experiments, QTL identification and gene expression analysis) to demonstrate how maternal genotype and phenotype (pod length) regulate an offspring phenotype (seed size). This maternal regulation model for seed weight is completely different from the previously proposed ones those act via ovule integuments and seed coat. Therefore, our study provides novel insights into the maternal control of seed weight and will open a new research field of seed weight in plants, especially in rapeseed.

## Experimental procedures

### Materials and field experiment

A large F_2_ population of 1150 individuals was derived from two sequenced rapeseed cultivars, Zhongshuang11 and No. 73290 (Huang *et al*., 2013). Of these, tens of F_2_ individuals with large or small seeds (thousand‐seed weight >5.5 or <3.5 g respectively) were directionally selected and self‐crossed for several generations, which produced 10 and 10 large and small‐seed RILs respectively (Figure S7). In addition, 192 F_2_ individuals were randomly selected and single‐seed descended for several generations, which produced 184 RILs named the BnaZNRIL population (Shi *et al*., 2015).

The BnaZNRIL population and the two parents were planted at Zhengzhou and Wuhan in 2011 and 2012 with a randomized complete block design with two replications. Each block contained three rows with 12 plants per row with a spacing of 33.3 × 16.7 cm. For each line, 10 representative individuals from the middle of each row in each block were harvested by hand at maturity. To directionally produce RILs with large or small seeds, selected lines were planted two rows with 12 plants per row with a spacing of 33.3 × 16.7 cm. In each generation, six representative individuals from the middle of each row were selfed at flowering and harvested at maturity. Moreover, the selected lines with large or small seeds were planted in twenty rows, each row having 10 plants with a spacing of 33.3 × 16.7 cm in 2012 (F_8_) at Wuhan for further systematic research.

The seeds were sown by hand, and the field management followed local standard agriculture practice.

### Microscopic analysis

Cell number and cell size of the outer layer of the seed coat and cotyledon of seeds were determined. Mature seeds were soaked in distilled water for 24 h and dissected to isolate the seed coat and embryo. Then, seed coat and embryo were fixed overnight with FAA solution (Tsukaya *et al*., 1993). The seed coat was rendered transparent by incubation overnight (12–24 h) in a chloral hydrate solution (Tsuge *et al*., 1996). After dehydration in an ethanol series (50%–70%–95%–100%), the embryo was infiltrated and subsequently embedded in paraffin wax (Hu *et al*., 2009). Sections were obtained using Leica RM 2016 microtome (Leica, Nanterre Cedex, France) and stained with safranin fast green. Observations were made with light fluorescence microscopy (IX‐71; Olympus, Tokyo, Japan). Cleared cells were photographed, and the mean cell area of at least 10 cells was determined based on 10 individuals using the Image J program (Bethesda, MD). The numbers of cells in the region of the outer seed coat and epidermis cotyledon were determined.

### Maternal effect and girdling experiment

The relative proportions of maternal and xenia effects were estimated by an improved genetic experiment of man‐made self‐ and reciprocal crosses (Wang *et al*., 2009), which leads to minimal environmental influence. For large‐seed line × small‐seed line cross, one large‐seed plant was selected as the maternal pollen acceptor, and half of the branches were self‐pollinated, while the other half were cross‐pollinated with the small‐seed line pollen. Reciprocal crosses were performed in the same way. Each cross was repeated six times, and pollination was completed within 1 day. A girdling experiment was performed by cutting with a blade around the green phloem in the middle of the pod stem (5 cm).

### The determination of carbohydrate content and photosynthetic activity

Sampled pod was separated into pod wall and seed gently on ice. Each fraction was transferred to plastic centrifuge tube in lipid nitrogen and stored at −80 °C. Total soluble sugar content and starch contents was analysed as described previously (Lunn and Hatch, 1995; Xu *et al*., 1997). Photosynthetic parameter was measured in the field using portable photosynthesis system (LI‐6400XT, LI‐COR, http://www.licor.com/). Measurement was always performed in the morning (9:00–11:00 AM). The measurement condition was the same with previous report (Hua *et al*., 2012). All data represented the means obtained from five plants for each RIL.

### Trait measurement

After harvest, the pod length and pod width (the maximum pod width) were measured based on 20 well‐developed pods randomly sampled from the main raceme. Pod number for the main raceme was counted. Matured seeds were threshed by hand from the open‐pollinated main raceme. The clean seeds were dried in the sun for at least 1 week. Next, seed number was counted, seed yield was weighed and seed diameter was measured. The other associated traits were calculated using the following equations. The pod surface area = −0.6 + 2.4 × (pod length × pod width), as described previously (Leng and Zhu, 1991); thousand‐seed weight = seed yield/seed number × 1000; seed number per pod = seed number/pod number; seed yield per pod = seed yield/pod number; seed surface area = 4π(seed diameter/2)^2^; seed volume = (4/3)π(seed diameter/2)^3^; bulk density = thousand‐seed weight/(seed volume × 1000). The seed oil and protein contents were determined using a Foss NIR Systems 5000 (Foss NIR Systems Inc., Maryland) as previously described (Hu *et al*., 2009).

### Quantitative genetic analyses

The broad‐sense heritability was calculated as *h* ^2^ = $\sigma_{g}^{2}$/($\sigma_{g}^{2}$ + $\sigma_{\mathrm{ge}}^{2}$/n+$\sigma_{e}^{2}$/nr), where $\sigma_{g}^{2}$ was the genetic variance, $\sigma_{\mathrm{ge}}^{2}$ was the interaction variance of the genotype with environment, $\sigma_{e}^{2}$ was the error variance, n was the number of environments and r was the number of replications. The estimation of $\sigma_{g}^{2}$, $\sigma_{\mathrm{ge}}^{2}$ and $\sigma_{e}^{2}$ was obtained from the SAS GLM procedure. Pearson's correlation coefficient and significant differences was calculated using SAS CORR and ANOVA procedures respectively.

Conditional phenotype values y_(T1/T2)_ was obtained by the mixed model approach for the conditional analysis of quantitative traits (Ma *et al*., 2005) using QGAStation1.0 (http://ibi.zju.edu.cn/software/qga/index.htm), where T1|T2 indicates that trait1 is conditioned by trait2. Conditional and unconditional QTL mapping was performed using the CIM program (Zeng, 1994) of the WinQTL cartographer2.5 software (http://statgen.ncsu.edu/qtlcart/WQTLCart.htm). A forward‐backward stepwise regression following model 6 was performed to choose the co‐factors (chosen with P_in_ = 0.05 and P_out_ = 0.05). The control marker number, window size and walking speed were set to 5, 10 and 1 cm respectively. A default genetic distance of 5 cm was used to define a QTL. The experiment‐wise LOD threshold was determined by a permutation test of 1000 repetitions (Churchill and Doerge, 1994). LOD score corresponding to *P* = 0.05 was used to identify significant QTLs (3.96–4.23 for thousand‐seed weight and 4.06–4.20 for pod length). To avoid missing QTLs with small effects, a lower LOD value corresponding to *P* ≤ 0.50 was adopted in the presence of suggestive QTLs (2.77–2.87 for thousand‐seed weight and 2.81–2.85 for pod length). The colocalized suggestive QTLs and all significant QTLs were admitted (Long *et al*., 2007).

### Solexa/Illumina sequencing and transcriptome analysis

Two pools (each consisting of ten RILs with large‐ or small‐seed) were analysed at the transcriptome level using Solexa/Illumina sequencing. Two pools’ RNA were isolated from pod wall (25‐DAF) and seed (25‐DAF, the time at which significant difference occurred between RILs with large‐ or small‐seed, Figure S8) which were mixed equivalently using a Plant Mini RNeasy Kit (Qiagen, Shanghai, China) respectively. Later, sample preparation was described in detail as previously (Xiang *et al*., 2010). After final cDNA library was synthesized, cDNA sample was sequenced by Illumina HiSeq^™^ 2000 (Illumina, San Diego, CA) to generate 125 nucleotide paired‐end sequence reads. Repetitive, low‐complexity and low‐quality raw reads were filtered out prior to assembly of sequence reads for later analysis. The resulting clean reads were mapped on *Brassica napus* genome and genes (Chalhoub *et al*., 2014), and functional categories were assigned on the basis of TAIR7 genome release (http://www.arabidopsis.org/) using SOAPaligner/soap2 (Li *et al*., 2009). Mismatches not more than 2 bp were allowed in the alignment.

The gene expression level was calculated using RPKM (Reads Per kb per Million reads) method (Mortazavi *et al*., 2008). A rigorous algorithm (Audic and Claverie, 1997) was used to identify DEGs. The FDR (false discovery rate) is used to determine the threshold of *P* value in multiple tests and analyses. In this study, FDR ≤ 0.001, and the absolute value of log_2_Ratio ≥ 1 were used to characterize DEGs.

For qRT‐PCR template, the reverse transcription reaction was performed using PrimeScript^™^II 1st Strand cDNA Synthesis Kit (Takara, Beijing, China). qRT‐PCR was performed using Bio‐Rad IQ5 with SYBR Green detection. Rapeseed β‐actin gene was used as internal control to normalize transcript levels. A cycling temperature of 57 °C and with a single peak on the melting curve to confirm the specificity of designed primer pairs, and each sample was assessed in triplicate.

## Conflict of interest

The authors have no conflict of interest to declare.

## Authors’ contributions

HZW, JQS and NL conceived and designed the experiments; NL, JQS, DJS and WP performed the experiments; NL and JQS analysed the data; HZW, JPZ, GHL and XFW contributed reagents/materials/analysis tools; NL and JQS wrote the manuscript. All the authors read and approved the final manuscript.

## Supporting information


**Figure S1** Distribution of thousand‐seed weight and pod length in RILs derived from the cross between Zhongshuang11 and No. 73290.

**Figure S2** Diagram of putative mechanistic relationships underlying the colocalization of QTLs for pod length and seed weight. (a) genetic linkage; (b) pleiotropy; (c,d) physiological interaction; (e,f) combination of pleiotropy and physiological interaction.

**Figure S3** qRT‐PCR validations of DEGs.

**Figure S4** Gene functional classification of clustered DEGs in pod wall. (a) normed to frequency in *Arabidopsis* set (±bootstrap SD); and (b) absolute values.

**Figure S5** Gene functional classification of clustered DEGs in seed. (a) normed to frequency in *Arabidopsis* set (±bootstrap SD); and (b) absolute values.

**Figure S6** The DEGs in part of the flavonoid branch in the phenylpropanoid pathway. Red arrows indicate *BnaA09g55530D* and other DEGs up‐regulated in corresponding location.

**Figure S7** Framework diagram for construction of RIL population.

**Figure S8** Fresh seed weight at different developmental stages.

**Table S1** Main agronomic and quality traits of the large‐ and small‐seed RILs.

**Table S2** Skeleton genetic linkage map for the RIL population.

**Table S3** Genome‐wide conditional and unconditional QTLs for thousand‐seed weight and pod length.

**Table S4** Statistics of reads produced by RNA‐seq of pod wall and seeds pooled from large‐ and small‐seed RILs.

**Table S5** Details of primers used for qRT‐PCR of selected genes.

**Table S6** (a) Details of the 631 DEGs identified in pod wall of two contrasting pools. (b) Functional classification of the 631 DEGs identified in the pod wall of two contrasting pools. (c) Fruit size genes collected from literature and websites.

**Table S7** (a) Details of the 486 DEGs identified in seeds of two contrasting pools. (b) Functional classification of the 486 DEGs identified in the seed of two contrasting pools. (c) Seed weight genes collected from literature and websites.

**Table S8** List of up‐regulated DEGs in seeds that are homologous to genes involved in development, cell division and nutrient reservoir activity.

**Table S9** (a) Significant KEGG pathways in pod wall of two contrasting pools. (b) Significant KEGG pathways in seed of two contrasting pools.
