# Supplementary figures and images for: Maternal control of seed weight in rapeseed (Brassica napus L.): the causal link between the size of pod (mother, source) and seed (offspring, sink)
Source: Plant Biotechnol J. 2018 Nov 28;17(4):736–49. doi: 10.1111/pbi.13011 (PMC6419582; doi:10.1111/pbi.13011)

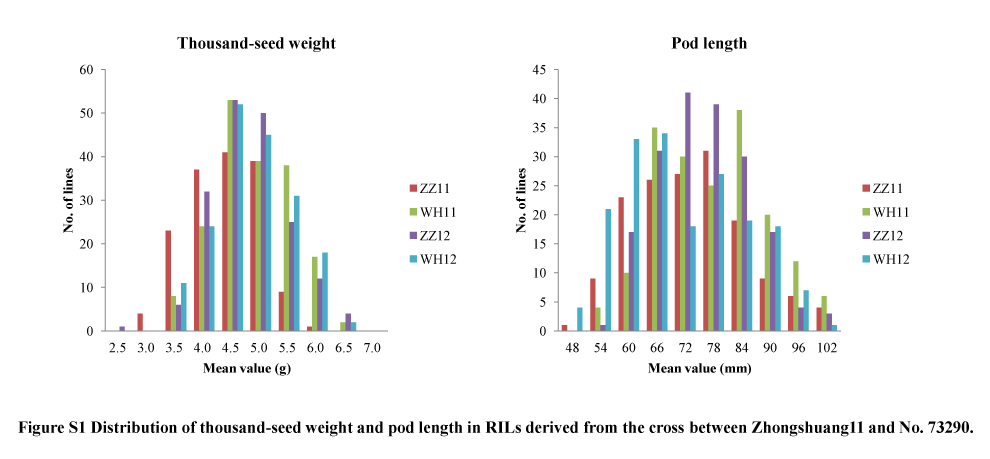

Supplement: Supplementary file 1 — Figure S1 Distribution of thousand‐seed weight and pod length in RILs derived from the cross between Zhongshuang11 and No. 73290. [file PBI-17-736-s001.tif]

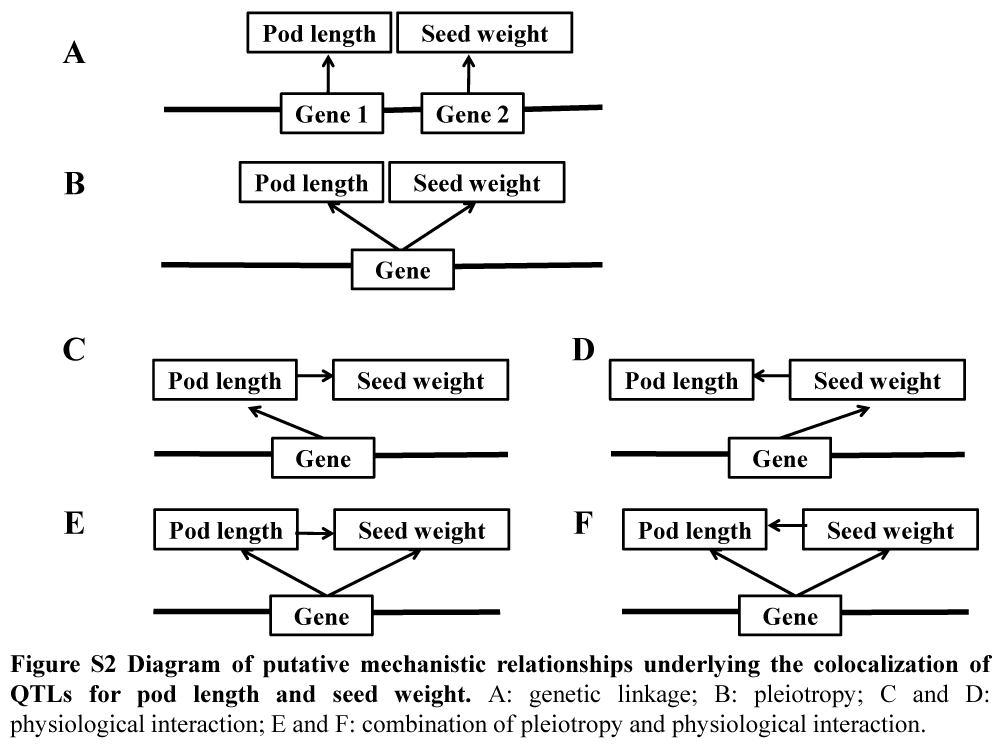

Supplement: Supplementary file 2 — Figure S2 Diagram of putative mechanistic relationships underlying the colocalization of QTLs for pod length and seed weight. (a) genetic linkage; (b) pleiotropy; (c,d) physiological interaction; (e,f) combination of pleiotropy and physiological interaction. [file PBI-17-736-s015.tif]

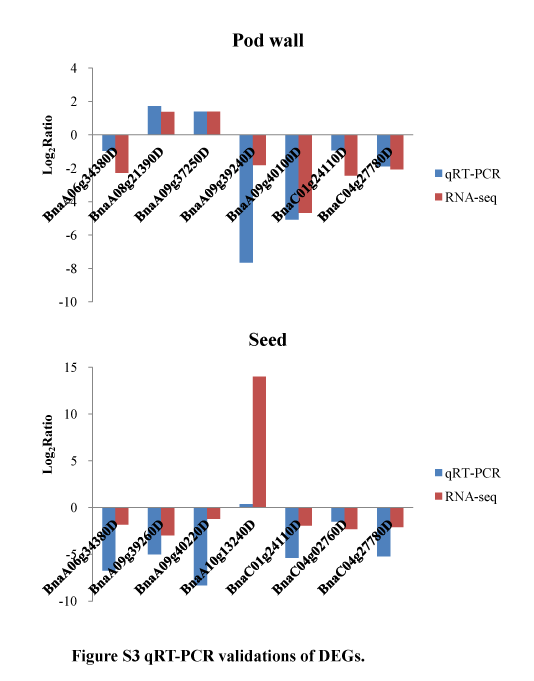

Supplement: Supplementary file 3 — Figure S3 qRT‐PCR validations of DEGs. [file PBI-17-736-s014.tif]

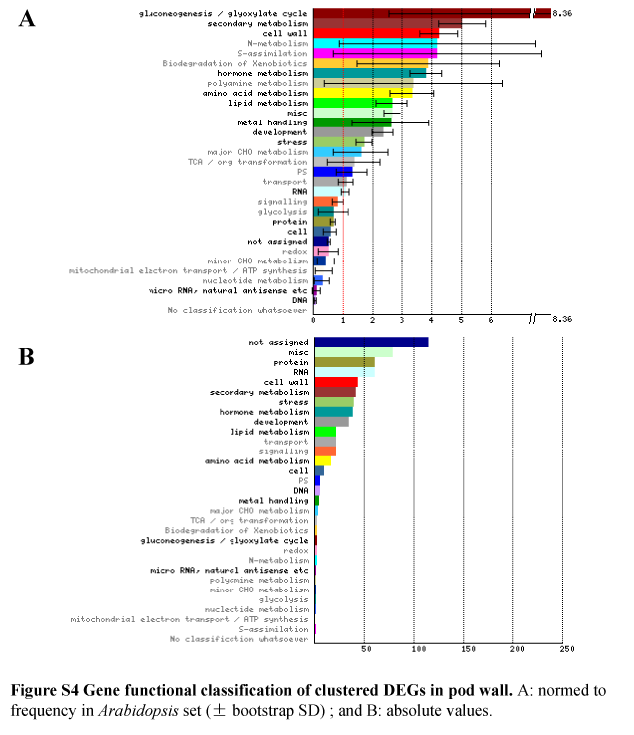

Supplement: Supplementary file 4 — Figure S4 Gene functional classification of clustered DEGs in pod wall. (a) normed to frequency in Arabidopsis set (±bootstrap SD); and (b) absolute values. [file PBI-17-736-s016.tif]

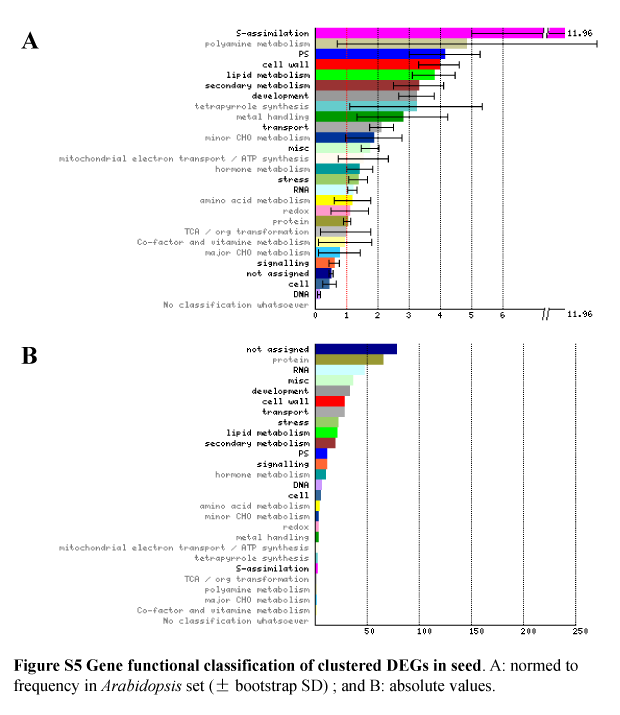

Supplement: Supplementary file 5 — Figure S5 Gene functional classification of clustered DEGs in seed. (a) normed to frequency in Arabidopsis set (±bootstrap SD); and (b) absolute values. [file PBI-17-736-s017.tif]

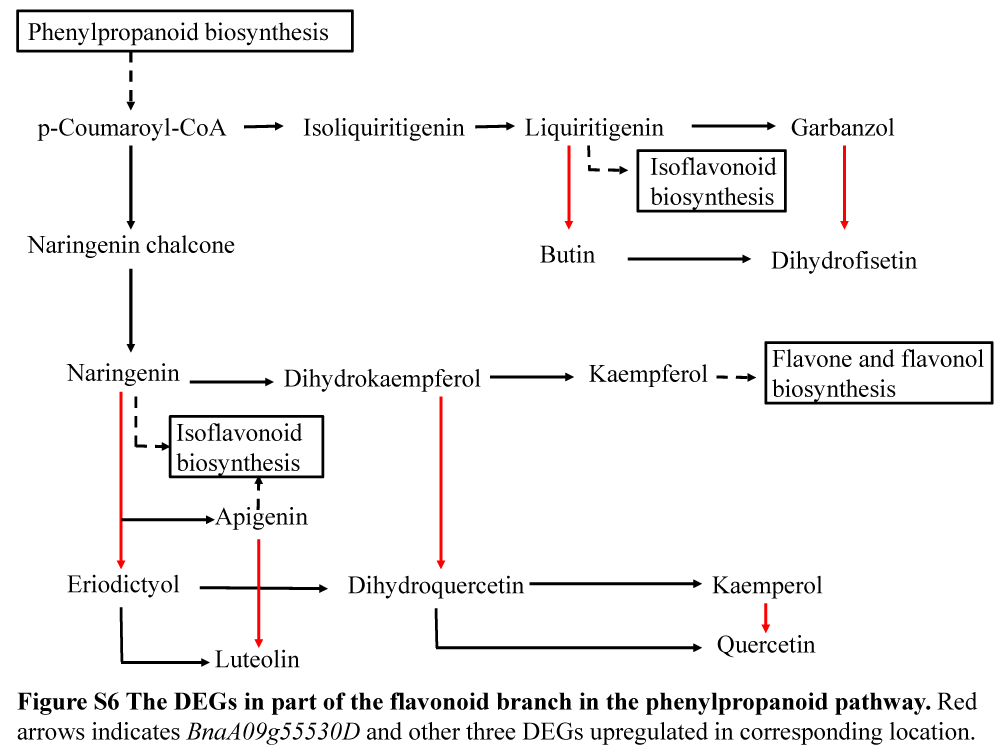

Supplement: Supplementary file 6 — Figure S6 The DEGs in part of the flavonoid branch in the phenylpropanoid pathway. Red arrows indicate BnaA09g55530D and other DEGs up‐regulated in corresponding location. [file PBI-17-736-s002.tif]

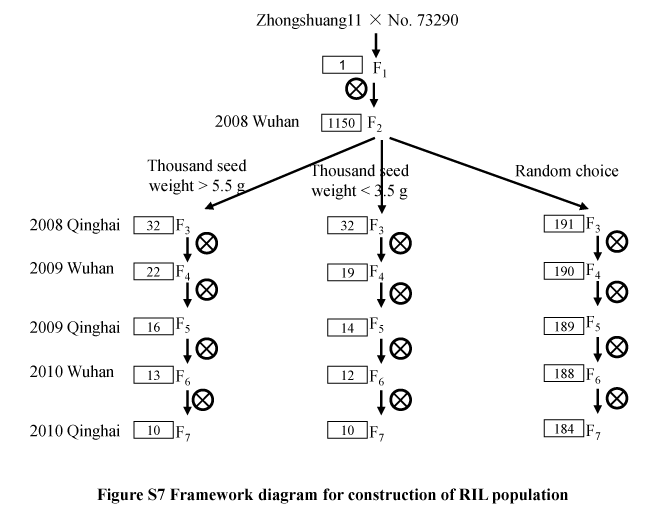

Supplement: Supplementary file 7 — Figure S7 Framework diagram for construction of RIL population. [file PBI-17-736-s003.tif]

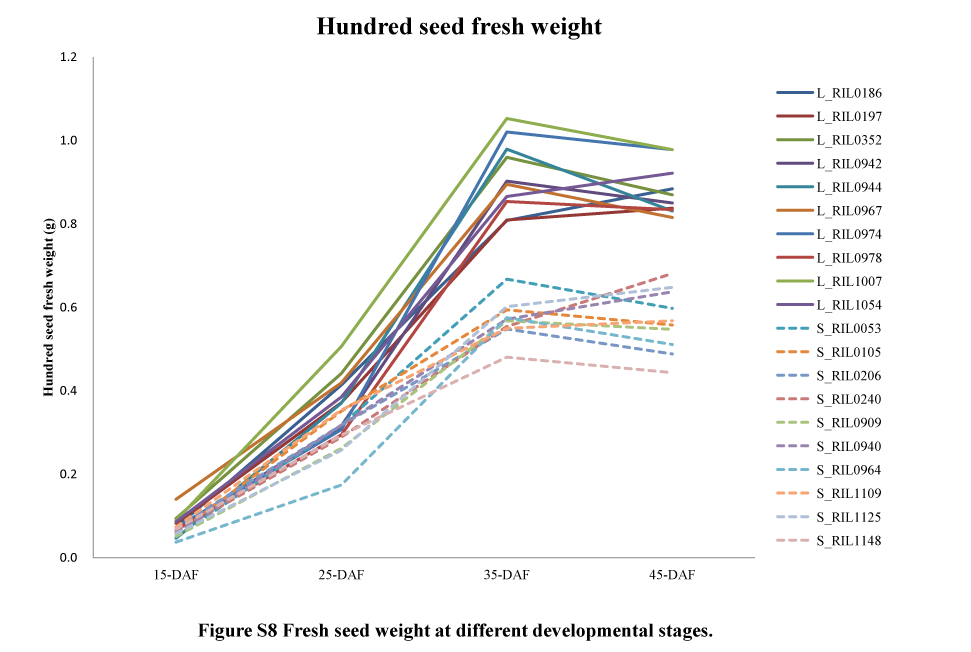

Supplement: Supplementary file 8 — Figure S8 Fresh seed weight at different developmental stages. [file PBI-17-736-s004.tif]
